# A case–control study on the effect of Apolipoprotein E genotypes on gastric cancer risk and progression

**DOI:** 10.1186/1471-2407-12-494

**Published:** 2012-10-25

**Authors:** Emma De Feo, Benedetto Simone, Roberto Persiani, Ferdinando Cananzi, Alberto Biondi, Dario Arzani, Rosarita Amore, Domenico D’Ugo, Gualtiero Ricciardi, Stefania Boccia

**Affiliations:** 1 Institute of Hygiene, Università Cattolica del Sacro Cuore, Rome, Italy; 2 Department of Surgery, Università Cattolica del Sacro Cuore, Rome, Italy; 3 IRCCS San Raffaele Pisana, Rome, Italy

**Keywords:** Gastric cancer, Apolipoprotein E, Genetic epidemiology, Polymorphism, Gene-environment interaction

## Abstract

**Background:**

Apolipoprotein E (ApoE) is a multifunctional protein playing both a key role in the metabolism of cholesterol and triglycerides, and in tissue repair and inflammation. The *ApoE* gene (19q13.2) has three major isoforms encoded by ε2, ε3 and ε4 alleles with the ε4 allele associated with hypercholesterolemia and the ε2 allele with the opposite effect. An inverse relationship between cholesterol levels and gastric cancer (GC) has been previously reported, although the relationship between a*poE* genotypes and GC has not been explored so far.

**Methods:**

One hundred and fifty-six gastric cancer cases and 444 hospital controls were genotyped for apoE polymorphism (ε2, ε3, ε4 alleles). The relationship between GC and putative risk factors was measured using the adjusted odds ratios (ORs) and their 95% confidence intervals (CIs) from logistic regression analysis. A gene-environment interaction analysis was performed. The effect of the apoE genotypes on survival from GC was explored by a Kaplan–Meier analysis and Cox proportional hazard regression model.

**Results:**

Subjects carrying at least one *apoE* ε2 allele have a significant 60% decrease of GC risk (OR=0.40, 95% CI: 0.19 – 0.84) compared with ε3 homozygotes. No significant interaction emerged between the ε4 or ε2 allele and environmental exposures, nor ε2 or ε4 alleles affected the median survival times, even after correcting for age, gender and stadium.

**Conclusions:**

Our study reports for the first time a protective effect of the ε2 allele against GC, that might be partly attributed to the higher antioxidant properties of ε2 compared with the ε3 or ε4 alleles. Given the study’s sample size, further studies are required to confirm our findings.

## Background

Apolipoprotein E (ApoE) is a small glycoprotein that plays a major role in the blood clearance of cholesterol-rich particles, known as remnant lipoproteins [1]. Besides its well-recognized role in lipid metabolism, ApoE has been shown to be involved in several pathophysiological processes, including antioxidant and immune activities, as well as a modulating effect on angiogenesis, tumor cell growth and metastasis induction [2]. The structural gene (19q13.2) for *apoE* is polymorphic with two single nucleotide polymorphisms (SNPs) within the coding region resulting in three different alleles (ε2, ε3, ε4) and six *apoE* genotypes (three homozygotes ε4/ε4, ε3/ε3 and ε2/ε2, and three heterozygotes ε4/ε3, ε3/ε2, ε4/ε2), each showing different receptor-binding abilities [3,4]. A meta-analysis reported a nearly linear relationship between *apoE* genotypes and the levels of total and LDL serum cholesterol (LDL-C) when the six genotypes are ordered as follows: ε2/ε2, ε2/ε3, ε2/ε4, ε3/ε3, ε3/ε4, ε4/ε4 [5]. In general, compared to the individuals with the ε3 allele, levels of total and LDL-C tend to be lower for those with the ε2 allele and higher for ε4 carriers [6]. In the past two decades, cross-sectional and prospective studies have reported that low serum cholesterol levels are associated with higher non-cardiovascular risk, especially cancer risk [7-10], thus, subjects with low serum total cholesterol levels are more likely to suffer from cancer. To date, the exact reason for such finding remains still unclear. Different explanations can be given as: (i) this association could theoretically reflect a direct causal role of cholesterol in cancer etiology, or it be due (ii) to some confounding factors that cause both low cholesterol and cancer, or (iii) to ‘reverse causation’, as low cholesterol levels could simply be the effect of cancer rather than the cause [11].

To fully answer the question whether a causal relationship exists between low cholesterol level and cancer, an alternative epidemiologic approach named “Mendelian randomization” can be used to overcome the problem of reverse causality and confounding. According to this approach, a genetic variation (e.g., *apoE*) that serves as a robust proxy for an environmentally modifiable exposure (e.g., serum cholesterol level) can be used in order to make causal inferences about a disease [12]. Benn et al. [13] have recently showed that low LDL-C were robustly associated with cancer in a large Danish cohort study, while a reduction in LDL caused by SNPs, including *apo E*, was not. By adopting the Mendelian Randomization approach, Benn concluded that his results are in accordance with those emerged from a cohort of elderly subjects treated with pravastatin [14] and from the Atherosclerosis Risk in communities cohort study [15], all suggesting low LDL are probably due to the preclinical cancer stage and *per se* do not cause cancer.

The role of *apoE* genotypes on gastric cancer (GC) aetiology has not been exlpored so far, as Benn et al. [13] considered all gastrointestinal cancer with no specific focus on GC. To date, four cohort studies [10,16-18] explored the relationship between serum cholesterol level and the development of GC. Among them, two Japanese cohort studies [10,18] and a Swedish study [16] reported that low serum cholesterol levels are independent risk factors for developing gastric cancer, especially the intestinal histotype. No association, however, was reported in a large Finnish cohort study [17]. Since the question of whether hypocholesterolemia is a predisposing factor for GC or a preclinical stage of GC has not been fully solved, our hospital-based case–control study aims to overcome this issue by directly looking at the relationship between *apoE* genotypes and GC as well as their interaction with potential effect modifiers.

## Results

General characteristics of the study population including 156 GC cases and 444 controls are presented in Table 1. Alcohol consumption was associated with an increased GC risk with ORs of 1.84 (95% CI = 1.10–3.07) and 3.29 (95% CI = 1.36–7.98) for moderate and heavy drinkers, respectively. A nearly doubled GC risk (OR=1.95, 95% CI: 1.06 – 3.60) was detected among individuals smoking more than 25 pack-years. In addition, family history of gastric cancer resulted to be associated with an increased GC risk (OR=3.14, 95% CI: 1.17 – 8.44; Table 1). Table 2 shows the distribution of the six *apo*E genotypes among GC cases and controls, with the ε3/ε2 genotype being less frequently represented in cases (9.87%) than in controls (15.67%).

**Table 1:** Odds ratios (95% CI) for gastric cancer according to selected variables and their frequency distribution among 156 gastric cancer cases and 444 controls

	**Cases**	**Controls**	**OR (95% CI) †**
	**n (%)**	**n (%)**	
Age (mean ± SD)	67.05±11.33	59.04±16.00	
Male gender	82 (53.2)	261 (58.8)	0.50 (0.30-0.83)
Alcohol drinkers			
0-6 g/day	60 (40.5)	251 (57.6)	1*
7-29 g/day	71 (48.0)	163 (37.4)	1.84 (1.10- 3.07)
>= 30 g/day	17 (11.5)	22 (5.0)	3.29 (1.36- 7.98)
Smoking status			
Never	78 (50.7)	238 (54.2)	1*
Ever	76 (49.3)	201 (45.8)	1.51 (0.94- 2.45)
Pack-years of smoking			
0	74 (51.7)	245 (57.0)	1*
1-25	31 (21.7)	111 (25.8)	1.40 (0.78- 2.50)
>25	38 (26.6)	74 (17.2)	1.95 (1.06- 3.60)
Fruit and vegetables intake			
High‡	41 (27.5)	116 (27.2)	1*
Low	108 (72.5)	310 (72.8)	1.18 (0.70- 1.98)
Grilled meat			
Low ^	106 (75.7)	314 (81.1)	1*
High	34 (24.3)	73 (18.9)	1.25 (0.72- 2.15)
Physical activity			
Any	29 (18.6)	93 (20.9)	1*
None	127 (81.4)	351 (79.1)	0.81 (0.45- 1.45)
Family history of cancer			
No	87 (62.2)	291 (71.2)	1*
Family history of gastric cancer	10 (7.1)	14 (3.4)	3.14 (1.17- 8.44)
Family history of other cancer	43 (30.7)	104 (25.4)	1.09 (0.66- 1.81)
ApoE allele frequency			
ε3	130 (85.5)	322 (80.1)	
ε2	8 (5.3)	39 (9.7)	
ε4	14 (9.2)	41 (10.2)	

† OR adjusted by age, gender, alcohol consumption (as continuous variable), packyears of smoking, grilled meat consumption and familiy history of cancer.

* reference category.

‡ at least three portions of fruit and vegetables per day.

^ less than four times/month.

**Table 2:** **Distribution of *ApoE *polymorphism among gastric cancer cases and controls**

	**Cases ‡**	**Controls^**	**All cases**	**Intestinal (n=79)**	**Diffuse (n=57)**
	**n (%)**	**n (%)**	**OR (95% CI)**^**†**^	**OR (95% CI)**^**†**^	**OR (95% CI)**^**†**^
ε3/ε3	109 (71.71)	253 (62.94)	1*	1*	1*
ε3/ε2	15 (9.87)	63 (15.67)	0.43 (0.21 -0.91)	0.34 (0.13 – 0.92)	0.57 (0.23 – 1.40)
ε3/ε4	27 (17.76)	75 (18.66)	0.70 (0.37 - 1.30)	0.75 (0.35 – 1.60)	0.64 (0.27 – 1.47)
ε2/ε2	0 (0.00)	5 (1.24)	NC	NC	NC
ε2/ε4	1 (0.66)	5 (1.24)	1.25 (0.10 - 15.10)	2.54 (0.21 – 31.17)	NC
ε4/ε4	0 (0.00)	1 (0.25)	NC	NC	NC
ε3/ε2 or ε2/ε2	15 (12.10)	68 (21.18)	0.40 (0.19 - 0.84)	0.31 (0.11 – 0.83)	0.53 (0.21 – 1.30)
ε3/ε4 or ε4/ε4	27 (19.85)	76 (23.10)	0.68 (0.36 - 1.26)	0.71 (0.34 – 1.53)	0.62 (0.27 – 1.43)

‡ Apolipoprotein genotype was measured in 152 cases.

^ Apolipoprotein genotype was measured in 402 controls.

† OR adjusted by age, gender, alcohol consumption (as continuous variable), packyears of smoking, grilled meat consumption and family history of gastric cancer.

* Reference category.

NC: not calculable due to few many values.

Frequency of *apoE* genotypes respected the Hardy-Weinberg Equilibrium (HWE) in the control group (*p*-value > 0.05, data not shown). From the multivariate analysis, individuals carrying at least one *apoE* ε2 allele had a significant 60% decreased risk of GC (OR=0.40, 95% CI: 0.19 – 0.84) when compared with those homozygous for the wild-type (ε3/ε3) (Table 2). When results were stratified according to tumour histology, the significant association between *apoE* ε2 allele carriers and gastric cancer appeared to be limited to the intestinal type, with an OR of 0.31 (95% CI: 0.11 – 0.83; Table 2). Quality controls showed 100% concordance between Restriction Fragment Length Polymorphism (RFLP) method and DNA sequencing.

Results of the gene-environment interaction analysis are presented in Table 3. No statistically significant interaction emerged between the ε4 or ε2 allele (*p-*value for interaction > 0.05), gender, age, alcohol consumption or fruit and vegetables intake.

**Table 3:** **Interactions between *apoE *genotypes and selected demographic and lifestyle variables on GC risk**

**Variables**	**Cases**	**Controls**	**Any *e2***	**Cases**	**Controls**	**Any *e4***
			**OR (95% CI)**^**†**^			**OR (95% CI)**^**†**^
**Gender**						
Female, *e3/e3*	51 (34.69)	96 (65.31)	1*	51 (34.69)	96 (65.31)	1*
Female, variant	8 (27.59)	21 (72.41)	0.58 (0.20 – 1.65)	12 (24.00)	38 (76.00)	0.55 (0.23 – 1.32)
Male, *e3/e3*	57 (26.64)	157 (73.36)	0.48 (0.27 – 0.88)	57 (26.64)	157 (73.36)	0.49 (0.27 - 0.89)
Male, variant	7 (12.96)	47 (87.04)	0.49 (0.11 – 2.18)	14 (26.92)	38 (73.08)	1.52 (0.44 – 5.27)
*P value for interaction°*			0.347			0.660
**Age**						
<60 years, *e3/e3*	25 (17.73)	116 (82.27)	1*	25 (17.73)	116 (82.27)	1*
<60 years, variant	4 (12.50)	28 (87.50)	0.35 (0.07 – 1.68)	7 (21.21)	26 (78.79)	0.70 (0.18 – 2.66)
≥60 years, *e3/e3*	84 (38.01)	137 (61.99)	2.85 (1.53 – 5.28)	84 (38.01)	137 (61.99)	2.84 (1.54 - 5.26)
≥60 years, variant	11 (21.57)	40 (78.43)	1.24 (0.21 – 7.26)	20 (28.57)	50 (71.43)	0.97 (0.21 – 4.36)
*P value for interaction*			0.807			0.964
**Alcohol drinking**						
Never, *e3/e3*	45 (24.06)	142 (75.94)	1*	45 (24.06)	142 (75.94)	1*
Never, variant	5 (12.50)	35 (87.50)	0.29 (0.08 – 1.04)	12 (18.18)	54 (81.82)	0.62 (0.28 – 1.37)
Ever, *e3/e3*	64 (36.57)	111 (63.43)	1.59 (0.88 – 2.86)	64 (36.57)	111 (63.43)	1.46 (0.81 – 2.62)
Ever, variant	10 (23.26)	33 (76.74)	1.78 (0.37 – 8.50)	15 (40.54)	22 (59.46)	1.28 (0.35 – 4.66)
*P value for interaction*			0.468			0.707
**Smoking status**						
Never, *e3/e3*	54 (29.03)	132 (70.97)	1*	54 (29.03)	132 (70.97)	1*
Never, variant	8 (20.51)	31 (79.49)	0.48 (0.18 – 1.28)	13 (20.00)	52 (80.00)	0.53 (0.23 – 1.23)
Ever, *e3/e3*	54 (31.40)	118 (68.60)	1.41 (0.80 – 2.50)	54 (31.40)	118 (68.60)	1.39 (0.79 – 2.45)
Ever, variant	7 (16.28)	36 (83.72)	0.68 (0.15 – 2.98)	13 (36.11)	23 (63.89)	1.98 (0.59 – 6.73)
*P value for interaction*			0.609			0.271
**Fruit and vegetables intake**						
Low, e3/e3	28 (29.47)	67 (70.53)	1*	28 (29.47)	67 (70.53)	1*
Low ,variant	2 (9.09)	20 (90.91)	0.14 (0.02 – 0.87)	9 (32.14)	19 (67.86)	0.84 (0.28 – 2.49)
High, e3/e3	78 (31.08)	173 (68.92)	1.17 (0.61 – 2.23)	78 (31.08)	173 (68.92)	1.12 (0.59 – 2.13)
High, variant	11 (19.30)	46 (80.70)	2.83 (0.38 – 21.05)	16 (22.86)	54 (77.14)	0.62 (0.16 – 2.37)
*P value for interaction*			0.309			0.487

† OR adjusted by age, gender, alcohol consumption (as continuous variable), packyears of smoking, grilled meat consumption and familiy history of gastric cancer.

* Reference category.

° By likelihood ratio test.

Median survival time from gastrectomy was 19 months with no statistical significant differences for *apoE* ε2 or ε4 allele carriers (*p-*value of log-rank test > 0.05), even when the analysis was restricted to 1 year and 2 years after surgical intervention (data not shown). Cox regression analysis showed no significant difference according to *apoE* after adjusting for age, gender, and stadium, even after stratifying by cancer histotype.

## Discussion

Our case–control study of 156 gastric cancer cases and 444 hospital-based controls evaluated for the first time the effect of *apoE* genotypes and their interactions with selected demographic and lifestyle factors on the risk of gastric cancer among an Italian population. According to our results, the *apoE* ε2 allele is associated with a 60% statistically significant decreased risk for GC when compared with the wild-type ε3 allele. This protective effect was particularly strong for the intestinal histotype. We were unable, however, to detect significant interactions between *apoE* alleles and lifestyle factors. Additionally, *apoE* genotypes do not appear to influence the survival time after surgical intervention, even when the analysis was restricted to the specific tumor histotypes.

Before interpreting our results, some limitations of the study should be taken into account. Firstly, on the basis of the prevalence of the *apoE* alleles in our control population, this study has *a priori* 90% power to detect an OR of 0.40 for the effect of the *apoE* ε2 allele (at 5% significance level). The study’s sample size limits the possibility to detect statistically significant gene–environment interactions, however, we need to increase the sample size in order to confirm our results. Secondly, data on serum cholesterol levels are not available in our study population, even though their utility would be limited in view of the fact such levels are affected by the cancer itself.

The effect of *apoE* genotypes has been previously investigated in relation to breast, colorectal, biliary tract, prostate, head and neck cancer, and haematological malignancies [19-24], with conflicting results. A recently published Mendelian randomization study addressed the unsolved question about the causal role of cholesterol in cancer etiology by examining if some SNPs including *apoE*, all linked to lifelong reduced plasma LDL-C, are causally related to an increased risk of cancer among two large Danish general population studies [13]. Results show an inverse relationship between cancer incidence or mortality and cholesterol levels, while no effect was demonstrated for the *apoE* alleles. Even with some limitations on the selected cohort, authors conclude that there is a substantial lack of causal effect of cholesterol on cancer risk including gastrointestinal cancers. Accordingly, the apparent contradiction between results relating plasma cholesterol levels and cancer risk with respect to *apo E*, might be indicative of a preclinical cancer stage involving the increased uptake of cholesterol from the blood for the cell growth and proliferation, thus lowering cholesterolemia prior to the clinical cancer diagnosis.

As for stomach cancer, two Japanese cohort studies [10,18] and a Swedish cohort study [16] reported a strong inverse association between serum cholesterol levels and risk of gastric cancer. Japanese studies, however, found the association far less stronger after exclusion of the early 3-year incident cases and advanced cases, thus suggesting the development of stomach cancer itself tends to lower total cholesterol levels. To overcome the issues related to reverse causation or confounding by lifestyle factors, we decided to clarify the role of cholesterol levels on GC risk by using a Mendelian randomization approach, namely by studying directly the effect of the *apoE* genotypes on a large series of Italian GC cases and controls. Our results show a statistically significant 60% decreased risk of GC associated with the ε2 allele.

Since ε2 carriers have a lower serum cholesterolemia than non- ε2 carriers, our finding contradicts the previously reported observation that low serum cholesterol levels increase GC risk [10,16,18]. ApoE, however, has many other functions beside its well-known role in lipid metabolism, that are potentially involved in cancer risk, as it is involved in tissue repair, inflammatory and immune response, cell growth and angiogenesis [2], and shows antioxidant properties [25]. Of importance, ApoE protein has certain antioxidative properties, with decreasing antioxidant activity in the order ε2 > ε3 > ε4 alleles [25]. Even if the molecular mechanisms responsible for the antioxidant properties of *apoE* is not clarified yet, a number of studies have examined the mechanisms through which *apoE* genotypes could affect the oxidative status-dependent mediators or biomarkers of oxidative stress [26-28]. *ApoE* ε2-carrier smoking individuals, who are exposed to nicotine, an important source of oxidative stress, have an almost 30% higher total antioxidant status compared with *apoE* ε3-carriers, measured as the capacity to inhibit the peroxidase-mediated formation of the 2,2-azino-bis-3-ethylbensthiazoline-6-sulfonic acid (ABTS+) radical, while *apoE* ε4 subjects show a 30% increased oxidised LDL [26].

Oxidative stress is given by an imbalance between increased production of reactive oxygen species and a significant decrease in the capability of antioxidant functions. The production of peroxides and free radicals associated with changes in the normal redox state of tissues can induce toxic effects including oxidative DNA damage that along with hypoxia, and acidosis, might be greatly involved in the pathogenesis of GC as it can be considered the cause as well as the consequence of tumor progression [29,30]. Some evidence showed a wide magnitude of oxidative stress in GC cases if compared with healthy individuals, as demonstrated by elevated levels of lipid peroxidation products and depletion of enzymatic and non-enzymatic antioxidants [31,32]. In view of all these findings, the results of our study, which reports a protective effect of the *apoE* ε2 allele on GC, might be explained by the improved antioxidant properties of ε2 allele compared with the ε3 or ε4 alleles, and this evidence can be especially true for GC, whose pathogenesis is strongly affected by smoking-related oxidative stress [33]. If our model holds true, we would expect an interaction between *apoE* ε2 allele and smoking status, however the limited power of our interaction analysis may have obscured it.

## Conclusions

In conclusion, our study provides for the first time evidence of a possible protective effect of the ε2 allele against GC. Further studies are required to confirm our results and to figure out if the protective effect is mediated through lowered cholesterol level or better antioxidant properties.

## Methods

### Study population

The study subjects were selected according to a case–control study design as previously described [34-36]. Briefly, cases were consecutive primary gastric adenocarcinoma patients, with histological confirmation, who underwent a curative gastrectomy in the "A. Gemelli" teaching hospital during the period 2002–2010. Controls were selected from cancer-free patients, with a broad range of diagnoses, admitted to the same hospital during the identical time period.

In closer details, about 50% of our control population is made of blood donors while the other half is made of patients undergoing surgical interventions as laparoscopic cholecystectomy or appendicitis or inguinal hernia and a smaller portion of patients affected by chronic diseases as hypertension or Chronic Obstructive Pulmonary Disease (COPD) undergoing periodical check-up.

All subjects were Caucasians born in Italy. The study sample size comprised 156 cases and 444 controls, with a participation rate of 98% among cases and 93% among controls. According to Lauren classification, the majority (58.1%) of gastric cancer cases were intestinal [37]. The tumours were located in the antrum (44.5%), in the corpus (14.8%), in the antrum/corpus (21.1%), in the cardia (3.1%), stumps (5.5%), in the fundum (1.6%), in the cardia/corpus (6.3%) and the entire stomach (3.1%). Based on the cytological and architectural atypisms, as well as the histo-pathological reports [38], patients’ tumours were classified accordingly: 70.0 % scarcely differentiated (G3), 27.6 % moderately differentiated (G2), 3.4 % well-differentiated (G1), while 51.7% were staged I-II and 48.3% staged III-IV.

Written informed consent was obtained from all study subjects, after which each subject provided a venous blood sample that was collected into EDTA-coated tubes. This study was performed according to the Declaration of Helsinki and was approved by the ethics committee of the Università Cattolica del Sacro Cuore.

#### Genotyping

DNA was extracted from the peripheral blood lymphocytes, and genotyping of *apoE* was performed using Restriction Fragment Length Polymorphism (RFLP). Briefly, 20ng of genomic DNA amplified using oligonucleotide primers 5’-TCC AAG GAG CTG CAG GCG GCG CA-3’ and 5’-GCC CCG GCC TGG TAC ACT GCC A-3’. Reactions were denatured for 3 minutes at 95°C, followed by 35 two-step cycles consisting of 10 seconds at 95°C then 10 seconds at 66°C. After the cycles were completed a final extension of 5 minutes at 95°C was performed. A 10 μl aliquot of each RFLP product was digested with 5 U of *Afl*III and a separate 10 μl aliquot was digested with 5 U of *Hae*II. Both *apoE* ε2 and *apoE* ε3 alleles were cut with *Afl*III to yield products of 50 and 168 bp, while the *apoE* ε4 allele remains uncut at 218 bp. Using *Hae*II, both *apoE* ε3and apoE ε4 alleles yield products of 23 and 195 bp, while the *apoE* ε2 allele remains uncut at 218 bp. The six possible genotypes were assigned by analyzing the patterns produced by the restriction digest. As for the quality controls, 5% of the samples were also sequenced, with standard DNA samples for each *apoE* genotype sent by Seripa et al. [39].

#### Data Collection

Cases and controls were interviewed by trained medical doctors using a structured questionnaire to collect information on demographic data, cigarette smoking, drinking history, dietary habits, physical activity and family history of cancer with a special focus on gastric cancer. Participants were asked to focus on the year prior to diagnosis (for controls the year prior to the interview date) when answering questions regarding lifestyle habits. Smoking status was categorized as never and ever-smokers (including both current and former smokers). Pack-years were calculated as years smoked multiplied by the current number (or previous number, for those who had quit) of cigarettes smoked per day divided by 20.

Fruit and vegetables intake was categorized as high if at least three portions of fruit and vegetables were consumed daily while grilled meat intake was defined as low if the consumption was less than 4 times/month. Family history of cancer referred to parents, siblings and offspring. Data concerning previous *Helicobacter pylori* infection were available only for gastric cancer cases. The response rate for completing the interview was 92% for cases and 97% for controls, with the exception of data relating to grilled meat intake (unknown in 10% of cases and 12.8% of controls) and the family history of cancer (unknown in 10% of cases and 8% of controls).

### Statistical analysis

The relationship between gastric cancer and putative risk factors were measured using the adjusted odds ratios (ORs) and their 95% confidence interval (CI) derived from logistic regression analysis using STATA software (version 10.0). Possible risk factors were considered to be confounders if the addition of that variable to the model changed the OR by 10% or more, and once a confounder of any estimated main effect was identified, it was kept in all models. Based on these criteria, we controlled for age, gender, alcohol and grilled meat consumption, cigarette smoking (pack-years) and family history of gastric cancer. A χ^2^-test of Hardy-Weinberg Equilibrium (HWE) for the three *apoE* alleles was performed among controls. In order to examine if the effect of the selected polymorphisms was modified by some environmental exposures, a stratified logistic regression analysis was performed, adjusting for the confounders previously identified. A gene-environment interaction analysis was performed by using those carrying the homozygous wild-type genotype (ε3/ε3 related to the *apoE* ε3 isoform) as the reference group. In this analysis, the genotypes were categorized as follows: presence of at least one *apoE* ε2 allele or presence of at least one *apoE* ε4 allele (the genotype ε2/ε4 was not included in either category), providing the other two *apoE* isoforms (*apo E*2 and *apo E*4).

In this analysis, age was categorized binomially (< 60 and ≥ 60 years old), smoking status was considered as ever/never cigarette smokers, and alcohol consumption as drinkers/non-drinkers (the latter including individuals whose alcohol intake was less than 7 g/day). In order to test for interaction between two exposure variables, the likelihood ratio test was used, with the individuals homozygous for wild-type genotype (ε3/ε3) and not exposed to the variables of interest used as the reference group.

Overall survival curves were calculated by the Kaplan-Meier product limit method from the date of diagnosis until death. If a patient was not dead, survival was censored at the time of the last visit. The log rank test was used to assess differences between subgroups. The risk of death related to ApoE isoforms was estimated by Cox's proportional hazards model. Hazard ratios (HR) were adjusted for age, gender, and stadium, with the wild-type genotype (ε3/ε3) as the reference group. In addition, analyses were stratified according to cancer histotype (intestinal/diffuse).

## Abbreviations

OR: Odds ratio; CI: Confidence interval; HWE: Hardy-Weinberg Equilibrium; ApoE: Apolipoprotein E; GC: Gastric cancer; SNPs: Single nucleotide polymorphisms.

## Competing interests

The authors declare they have no competing interests.

## Authors’ contributions

EDF, WR and SB conceived the study and implemented the final draft of the manuscript; EDF and BS performed the statistical analysis and wrote the paper; RP, AB, FC and DDU recruited gastric cancer cases and controls; DA and RA processed blood samples and genotyped for *apoE*. All authors read and approved the final manuscript.

## Source of support

No funding support to be declared.

## Pre-publication history

The pre-publication history for this paper can be accessed here:

http://www.biomedcentral.com/1471-2407/12/494/prepub
